# Rapid and robust on‐scene detection of cocaine in street samples using a handheld near‐infrared spectrometer and machine learning algorithms

**DOI:** 10.1002/dta.2895

**Published:** 2020-07-27

**Authors:** Ruben F. Kranenburg, Joshka Verduin, Yannick Weesepoel, Martin Alewijn, Marcel Heerschop, Ger Koomen, Peter Keizers, Frank Bakker, Fionn Wallace, Annette van Esch, Annemieke Hulsbergen, Arian C. van Asten

**Affiliations:** ^1^ Dutch National Police, Unit Amsterdam, Forensic Laboratory Amsterdam the Netherlands; ^2^ Van't Hoff Institute for Molecular Sciences University of Amsterdam Amsterdam the Netherlands; ^3^ Wageningen Food Safety Research part of Wageningen University and Research Wageningen the Netherlands; ^4^ Dutch Customs Laboratory Amsterdam the Netherlands; ^5^ National Institute of Public Health and the Environment (RIVM) Bilthoven the Netherlands; ^6^ Netherlands Forensic Institute (NFI) Den Haag the Netherlands; ^7^ Co van Ledden Hulsebosch Center (CLHC), Amsterdam Center for Forensic Science and Medicine Amsterdam the Netherlands

**Keywords:** cocaine, forensic illicit‐drug analysis, indicative testing, *k*‐nearest neighbors, near‐infrared

## Abstract

On‐scene drug detection is an increasingly significant challenge due to the fast‐changing drug market as well as the risk of exposure to potent drug substances. Conventional colorimetric cocaine tests involve handling of the unknown material and are prone to false‐positive reactions on common pharmaceuticals used as cutting agents. This study demonstrates the novel application of 740–1070 nm small‐wavelength‐range near‐infrared (NIR) spectroscopy to confidently detect cocaine in case samples. Multistage machine learning algorithms are used to exploit the limited spectral features and predict not only the presence of cocaine but also the concentration and sample composition. A model based on more than 10,000 spectra from case samples yielded 97% true‐positive and 98% true‐negative results. The practical applicability is shown in more than 100 case samples not included in the model design. One of the most exciting aspects of this on‐scene approach is that the model can almost instantly adapt to changes in the illicit‐drug market by updating metadata with results from subsequent confirmatory laboratory analyses. These results demonstrate that advanced machine learning strategies applied on limited‐range NIR spectra from economic handheld sensors can be a valuable procedure for rapid on‐site detection of illicit substances by investigating officers. In addition to forensics, this interesting approach could be beneficial for screening and classification applications in the pharmaceutical, food‐safety, and environmental domains.

## Graphical abstract

The novel application of 740‐1070 nm small wavelength range NIR spectroscopy to confidently detect cocaine in case samples is demonstrated. Multi‐stage machine learning algorithms are applied to exploit the limited spectral features and predict not only the presence of cocaine but also predict a concentration and sample composition. A model based on >10,000 spectra from case samples yielded 97% true positive and 98% true negative results. The practical applicability is shown on over 100 case samples not included in model design.

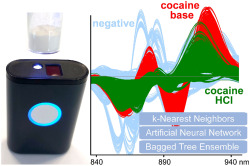

## 1 INTRODUCTION

Cocaine is one of the most abundant drugs of abuse worldwide, with an estimated global annual production of ~2000 metric tons of pure cocaine.^1^ Even though the recreative use of this drug has been banned for years, cocaine abuse is still increasing. In 2020, the annual report of the Drugs Information and Monitoring System in the Netherlands revealed that around 6.5% of the Dutch population has used cocaine at least once.^2^ In 2018, more than 40 metric tons of cocaine was confiscated in the Netherlands alone. Cocaine street samples are becoming increasingly more potent, with cocaine contents in the Netherlands increasing from an average of 48.7 wt% in 2011 to 65.5 wt% in 2018.^2^ These percentages are consistent with cocaine contents reported throughout Europe, with averages of 51–73 wt% between countries with an interquartile range of 40–84 wt%.^3^

In addition to cocaine, the global illicit‐drug market consists of many other substances such as the conventional synthetic drugs amphetamine, methamphetamine, and 3,4‐methylenedioxymethamphetamine (MDMA) as well as many new psychoactive substances (NPSs) and precursor chemicals that can be controlled depending on national legislation. The great variety of substances is not the only challenge when developing suitable indicative tests for drug‐suspected seizures; street samples are often not pure, because they are regularly adulterated with cutting agents. These cutting agents occur in great variety, and common adulterants for cocaine include sugars (eg*,* mannitol, inositol), caffeine, phenacetin, lidocaine, procaine, paracetamol, and levamisole.^2^, ^4^, ^5^, ^6^ The ever‐changing and complex illegal market makes it a difficult task for law enforcement to control these substances. Therefore, there is a need for reliable, affordable, and fast detection techniques to identify the suspected compounds.

Conventionally, simple colorimetric tests are used to obtain a first indication of whether a substance contains a frequently occurring illicit compound.^4^, ^7^ The most commonly used indicative test for cocaine in seized material is the Scott or Ruybal test that produces a blue color from a cobalt(II)thiocyanate complex formed in the presence of cocaine.^8^, ^9^ In addition to test solutions prepared in the forensic laboratory, a large variety of commercial test kits based on this complex are manufactured in the form of pouches, ampules, swabs, and wipes.^10^ A well‐known limitation of these cobalt(II)thiocyanate‐based tests is their false‐positive (FP) response to several common adulterants such as levamisole and lidocaine.^11^ This is a major drawback as levamisole is one of the most frequently used cutting agents, present in more than 40% of seized cocaine samples.^12^ It is therefore not unlikely to encounter pure levamisole, or another legal yet FP substance, in a drug‐related setting, either as a pure cutting agent or packed in wrappers to be sold as cocaine in a scam. Another limitation of colorimetric tests is their limited specificity. These tests are available only for a limited number of traditional illicit substances, and each specific test formulation has its own profile of FP reactions and thus needs a dedicated validation study. In addition, colorimetric tests require touching and manipulating the sample and thus cause a potential safety risk for the investigating officer when highly potent substances such as fentanyl and its derivatives are encountered.^13^, ^14^ In the forensic field, colorimetric tests are accepted to provide a presumptive result suitable to detain a suspect and to select samples for confirmatory laboratory analysis. Only the laboratory results are subsequently used as evidence in court by the prosecution.^15^ Therefore, confiscated samples always require additional testing in a forensic laboratory to employ more advanced analyses, including gas chromatography–mass spectrometry (GC–MS), liquid chromatography–MS, or Fourier‐transform infrared (FT‐IR) spectroscopy.

Alternative rapid and portable techniques that can overcome the limitations of colorimetric tests are being explored. Technical innovations in attenuated total reflectance (ATR)‐FT‐IR spectroscopy led to the development of portable ATR‐FT‐IR devices to provide on‐scene analysis of samples.^16^ Also, electrochemical tests that can overcome the specificity issues of the Scott test have been developed.^11^, ^17^ However, both electrochemical tests and ATR‐FT‐IR spectroscopy still require touching and handling of sample material. Raman spectroscopy and near‐infrared (NIR) spectroscopy are two techniques that can analyze through the packaging material without handling the sample and can be operated by minimally trained staff. These techniques therefore provide an intrinsic safer procedure for the operator.^16^, ^18^, ^19^ Although commercial Raman‐handheld spectrometers are already being used by law enforcement officers, this technique still faces limitations. One of the major problems is that fluorescent compounds interfere and obscure Raman signals, leading to limits of detection that are dependent on the specific adulterants present in the sample.^20^ In addition, because commercial Raman devices possess built‐in library‐based techniques, they cannot always detect low concentrations of controlled substances in mixtures nor can they detect compounds that are not included in the library.

In contrast, NIR analyzers are not affected by fluorescence and are much cheaper and smaller than Raman devices. NIR analyzers therefore have the potential to be implemented as cost‐effective standard equipment for the general police or customs officers, whereas the more‐expensive Raman instruments more likely remain a tool for more‐specialistic forensic investigators for economic reasons and the expertise required to interpret the measurements correctly. An example of a commercial NIR spectrometer is the SCiO from Consumer Physics (Herzliya, Israel). The SCiO operates in a narrow‐wavelength range (740–1070 nm or 13,500–9350 cm^−1^) unlike many other NIR spectrometers operating in higher‐wavelength ranges up to 2500 nm (4000 cm^−1^). The SCiO scanner is however one of the cheapest devices that are currently commercially available. NIR spectra are based on vibrational overtones and combination bands, yielding raw spectra that are initially noninformative.^21^ Therefore, extensive data preprocessing followed by chemometric data modeling is needed to extract useful information from the data. Several studies have already shown that chemometric analysis of the data is of great use to apply NIR devices operating at longer‐wavelength ranges in forensic casework.^22^, ^23^, ^24^ Liu et al^22^ successfully demonstrated the benefits of a multimodel approach on forensic drug samples. They used soft independent modeling of class analogy (SIMCA) for the classification of spectra with a methamphetamine, ketamine, heroin, or cocaine class followed by individual partial least square (PLS) regression models for quantification. Hespanhol et al^23^ were the first to apply different models on NIR spectra to answer a set of forensic‐relevant questions, including SIMCA for cocaine HCl and cocaine base classification, PLS for quantitative information, and multivariate curve resolution for establishing the degree of adulteration. Both earlier studies used NIR devices with relatively extensive wavelength ranges, that is, 1000–2500 nm^22^ and 900–1700 nm depending on the choice of the NIR equipment.^23^ NIR spectrometers operating at wavelength ranges above 1000 nm require more advanced light sources, active cooling in some cases, and more expert application knowledge and are thus less economically attractive when considering the large‐scale use in the field by law enforcement officers. To our knowledge, no forensic studies have been published on the applicability of low‐cost NIR spectrometers operating in the relatively limited spectral region of 740–1070 nm.

Chemometric data analysis approaches are widely used in analytical chemistry and forensic science in disciplines dealing with large amounts of data of high complexity or with limited spectral features.^25^, ^26^ The frequently used classification schemes include SIMCA, principal component analysis (PCA), linear discriminant analysis (LDA), and partial least squares‐discriminant analysis (PLS‐DA), whereas partial least squares‐regression (PLS‐R) is commonly used to fit a linear correlation between the multidimensional spectral data and the concentration of a compound of interest.^27^, ^28^ In addition, approaches such as support vector machines (SVM), k‐nearest neighbors (kNN), random forest, and artificial neural networks (ANNs) already proved their value for NIR spectral data modeling outside of the forensic field.^29^ Classification algorithms based on spectroscopic data are operational in the fields of food and medicine authentication. Teye et al^30^ demonstrated the correct classification of the origin and quality of rice by both kNN and SVM machine learning models applied on short‐range (740–1070 nm) NIR spectra after multiplicative scatter correction as preprocessing and PCA for data dimensionality reduction. Another example is the detection of counterfeit tablets among genuine pharmaceutical products for several active pharmaceutical ingredients.^31^ In this study, both a short‐wavelength SCiO and a longer‐wavelength NIR device were used. A machine learning approach using SVM showed better performance on their SCiO data than a conventional LDA approach.^31^ Jiménez‐Romero et al demonstrated the classification potential of both PLS‐DA and kNN on 1000–2500 nm NIR spectra to compare the production batches of pharmaceuticals.^32^ ANNs are another machine learning approach that has successfully been applied on spectral data for classification purposes.^33^, ^34^ ANN often proved superior compared to SVM and PLS‐DA approaches.^29^, ^33^ An example of ANNs applied on NIR data includes cellulose pulp dryness determination in industrial processing.^29^ A limitation of ANN lies within the nature of the self‐learning algorithms causing small but inevitable variations between iterations of model development, which results in some variation in model performance. Strategies to overcome this limitation and further enhance the classification power are repetitive or multistage models, called ensembles.

This study demonstrated the applicability of the low‐cost, small‐wavelength NIR spectrometer on a large set of cocaine‐containing street samples as well as negative samples containing (mixtures of) adulterants and other illicit drugs frequently encountered in forensic casework. The samples were analyzed by 15 individual (SCiO) NIR scanners to explore the robustness of the device and the potential of large‐scale scanner use in combination with a central model for data interpretation. This resulted in a data set of more than 10,000 individual measurements. All street samples were representative of the Dutch cocaine market, as they originated from seized material provided by the Netherlands Forensic Institute (NFI), the Dutch Customs, and the Dutch National Police. Multiple successive chemometric models and machine learning algorithms were used to (a) characterize and (b) determine the composition of these samples. The model includes an adjustable weight percent‐cocaine threshold that can be used to optimize the FP versus false‐negative (FN) classification rates for specific forensic and security situations. Because the average purity of cocaine in street samples usually exceeds 40 wt%,^2^, ^3^, ^5^ a threshold below this level can safely be set for optimal accuracy (in this study 20 wt%). Thus, the developed NIR approach resulted in a reliable, adjustable, rapid, and affordable presumptive test for cocaine in seized powder samples with 98.2% correct classification of cocaine‐negative spectra and 97.2% correct classification of cocaine‐positive spectra.

## 2 MATERIALS AND METHODS

### 2.1 Chemicals and reagents

Four sets of samples were incorporated in this study. Three sets were used for model design and cross‐validation: 90 cocaine case samples, including both cocaine HCl and cocaine base (Set A), 40 negative samples (Set B), and 50 cocaine reference samples at various concentrations (Set REF). One set consisting of 76 case samples and 33 standards was used only for external validation.

All 90 cocaine‐containing case samples in Set A were provided by the NFI and originated from material seized by the Dutch National Police in 2017. The 40 samples consisting of cutting agents, adulterants, and other drug samples in Set B were obtained from the following providers: USP‐grade acetaminophen (paracetamol), benzocaine (98%), diltiazem (99%), reagent‐grade glucose, *myo*‐inositol (99%), and levamisole (99%) were from Sigma‐Aldrich (St. Louis, MO, USA). Acetylsalicylic acid (Ph.Eur.), boric acid (p.a.), and procaine (Ph.Eur.) were obtained from Merck (Darmstadt, Germany). Ascorbic acid (vitamin C, >99.5%) was obtained from Fluka (Seelze, Germany). Research‐grade phenacetin was obtained from Brunschwig (Amsterdam, the Netherlands); caffeine (Ph.Eur.) from Brocacef B.V. (Maarssen, the Netherlands); lidocaine (Ph.Eur.) from Applichem (Darmstadt); and promethazine HCl from Acros Organics (Geel, Belgium). Lactose, mannitol, nondairy creamer, saccharose (confectioner's sugar), and wheat flour were purchased at local grocery stores. Smartshop blend mix of caffeine:lactose:mannitol was a seized case sample by the Amsterdam police. The non‐cocaine drugs‐of‐abuse samples—MDMA powder, 4‐fluoroamphetaminetablets, amphetamine, diazepam 10 mg tablets, flunitrazepam 2 mg tablets, ketamine, mephedrone, methamphetamine crystals, methylphenidate HCl 10 mg tablets, oxazepam 50 mg tablets, and sildenafil citrate 100 mg tablets—were seized case samples provided by the Amsterdam police.

Non‐drug‐containing mixtures of acetaminophen:caffeine, acetaminophen:phenacetin, levamisole:acetaminophen:lidocaine, levamisole:lidocaine, levamisole:phenacetin, levamisole:phenacetin:procaine, phenacetin:lidocaine, and phenacetin:procaine were prepared by grinding and mixing equal‐weight aliquots of the aforementioned compounds in a mortar.

The 50 diluted reference samples (Set REF) with known cocaine HCl content were provided by the Dutch Customs Laboratory. This set consisted of cocaine HCl (*cocaini hydrochloridum* Ph. Eur, obtained from Duchefa Farma, Haarlem, the Netherlands) and mixtures of this sample at the given percentages. Note that these percentages reflect the cocaine HCl content as weight percentage and not the corrected cocaine base levels. Set REF contents: 100% cocaine HCl; 81%, 76%, 44%, 33%, 17%, 9%, and 0% cocaine HCl in lactose; 73%, 50%, 35%, 21%, 13%, 5%, and 0% cocaine HCl in phenacetin; 70%, 50%, 35%, 25%, 20%, 15%, 11%, 5%, and 0% cocaine HCl in glutamine; 50%, 26%, 17%, and 0% cocaine HCl in procaine; 70%, 50%, 35%, 21%, 10%, 5%, and 0% cocaine HCl in ascorbic acid (vitamin C); 48%, 26%, 17%, 10%, 6%, and 0% cocaine HCl in acetaminophen; 42%, 34%, 30%, 25%, 20%, 19%, 10%, 5%, and 0% cocaine HCl in caffeine.

The 76 external validation samples were case materials seized by the Amsterdam police between October 2019 and March 2020. The 33 validation standards were inositol, levamisole, and caffeine, each mixed with cocaine HCl from 0% to100% in 10% intervals.

The nature of the case samples was established using the accredited GC–MS analysis at the NFI, the Dutch Customs Laboratory, or the Amsterdam Police Laboratory. Cocaine concentration and type (HCl or base) for the samples of Set A were available from GC‐Flame Ionization Detection and precipitation test results. Both these qualitative and quantitative analyses were performed using the routine procedures and validated methods embedded in the ISO 17025 accredited quality frameworks maintained by these three institutes. The characteristics of the Set A cocaine case samples were as follows: 58× cocaine HCl (ranging from 85.5% to19.1%, average 64.4%) and 32× cocaine base (ranging from 99.4% to 31.5%, average 75.4%).

Non‐cocaine samples in tablet, rock, or crystal form were ground in a mortar to obtain a powder. Coarse powdered case samples were not ground further. All individual samples were transferred to clear borosilicate glass vials (4 mL, 15 mm diameter × 48 mm height) from VWR (Amsterdam, the Netherlands). All vials used for model development were filled with at least 5 mm of powder to ensure a sufficient sample layer for diffuse reflectance spectroscopy.

### 2.2 Instruments and settings

**FIGURE 1:**
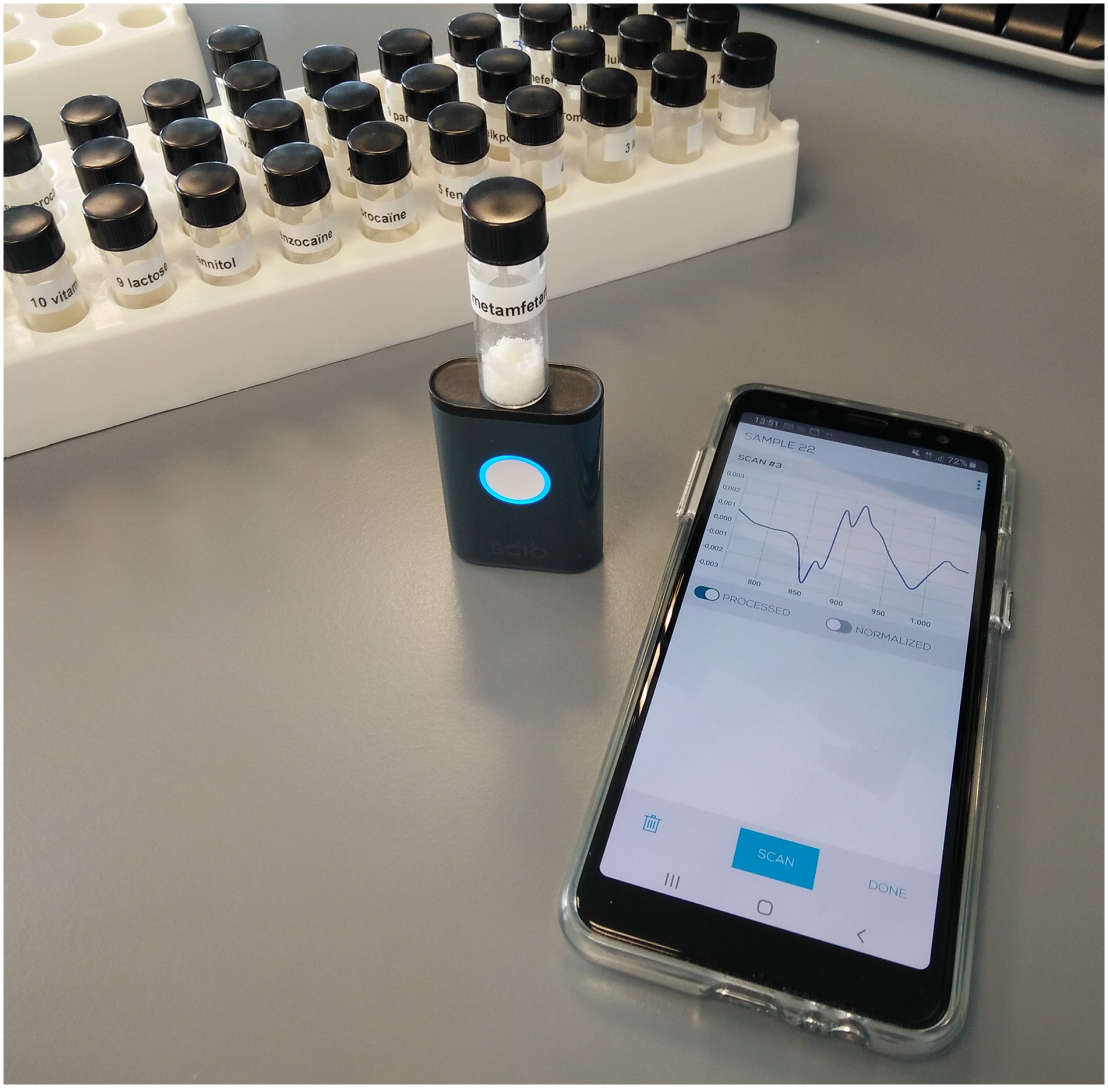
Scanning procedure for SCiO near‐infrared sensor with the glass sample vial placed directly on top of the light source and detection window

NIR spectra were recorded using a pocket size (54 × 36 × 15 mm, 35 g) SCiO handheld NIR spectrometer from Consumer Physics, hardware version 1.2. All SCiO sensors were operated via the SCiO “The Lab” mobile application on the operator's iOS or Android smartphone or tablet using a Bluetooth connection. Before use, each sensor was calibrated using the built‐in calibration device in the sensor cover. The sample scanning procedure is shown in Figure 1: the sensor was positioned with the detector side facing upward; the sample‐containing vial was placed directly on top of the sensor in such a way that both the NIR light source and the detector window are obscured; the scan was started from the connected mobile device. Typical scanning and processing times took several seconds. The standard spectral range of the sensor was 740–1070 nm, and no additional settings (ie, exposure time, spectrum averaging) could be optimized. Data were stored in The Lab cloud environment, and raw spectral data were exported for further data analysis. The exported NIR spectra contained 331 individual variables. No information regarding the spectral resolution of the device was provided by the manufacturer. However, associated patents suggest a wavelength‐dependent resolution between 5 and 20 nm in combination with data splicing to obtain a data set at 1 nm resolution. A total of 15 individual SCiO devices were used: 13 for the Set A and Set B experiments and 6 for the Set REF experiments, with 4 pocket scanners used within all sets. For each SCiO, at least five replicate scans were recorded per sample. For the 180 samples, this resulted in a total data set of 10,059 individual NIR spectra used for the model. The 109 external validation samples were scanned fivefold on a single device, thus leading to a set of 545 scans. Results for these scans were predicted by the model, but these scans were not involved in model design and optimization.

### 2.3 Data analysis

Raw spectral data were imported in Unscrambler 11 (Camo Analytics, Oslo, Norway) for data preprocessing optimization and exploratory analysis using PCA, SIMCA, and PLS‐R. Preliminary models based on SIMCA–PLS‐R were found to produce reasonable results although some disruptive FP results were obtained for a few common cutting agents. Subsequent model building was performed in R version 3.6.3 (R Foundation for Statistical Computing, Vienna, Austria)^35^ using RStudio version 1.2.5033. R‐packages, prospectr_0.2.0,^36^ signal_0.7‐6,^37^ and caret_6.0‐86,^38^ were used.

The following two data preprocessing methods were found suitable based on visual inspection of the data: standard normal variate (SNV)^36^ preprocessing followed by either a first‐ or second‐order derivative with Savitzky–Golay^37^ smoothing using a 19‐datapoint window. This window size was optimal for noise removal, as shown in Figure S1 (supporting information). When indicated that data are processed with focus on a specific region of interest (ROI), the following ROIs were used: for first‐order derivative data: the 839–939 nm part of the spectrum; for second‐order derivative data: the 839–914 nm part of the spectrum.

## 3 RESULTS AND DISCUSSION

### 3.1 Spectral reproducibility and selectivity

**FIGURE 2:**
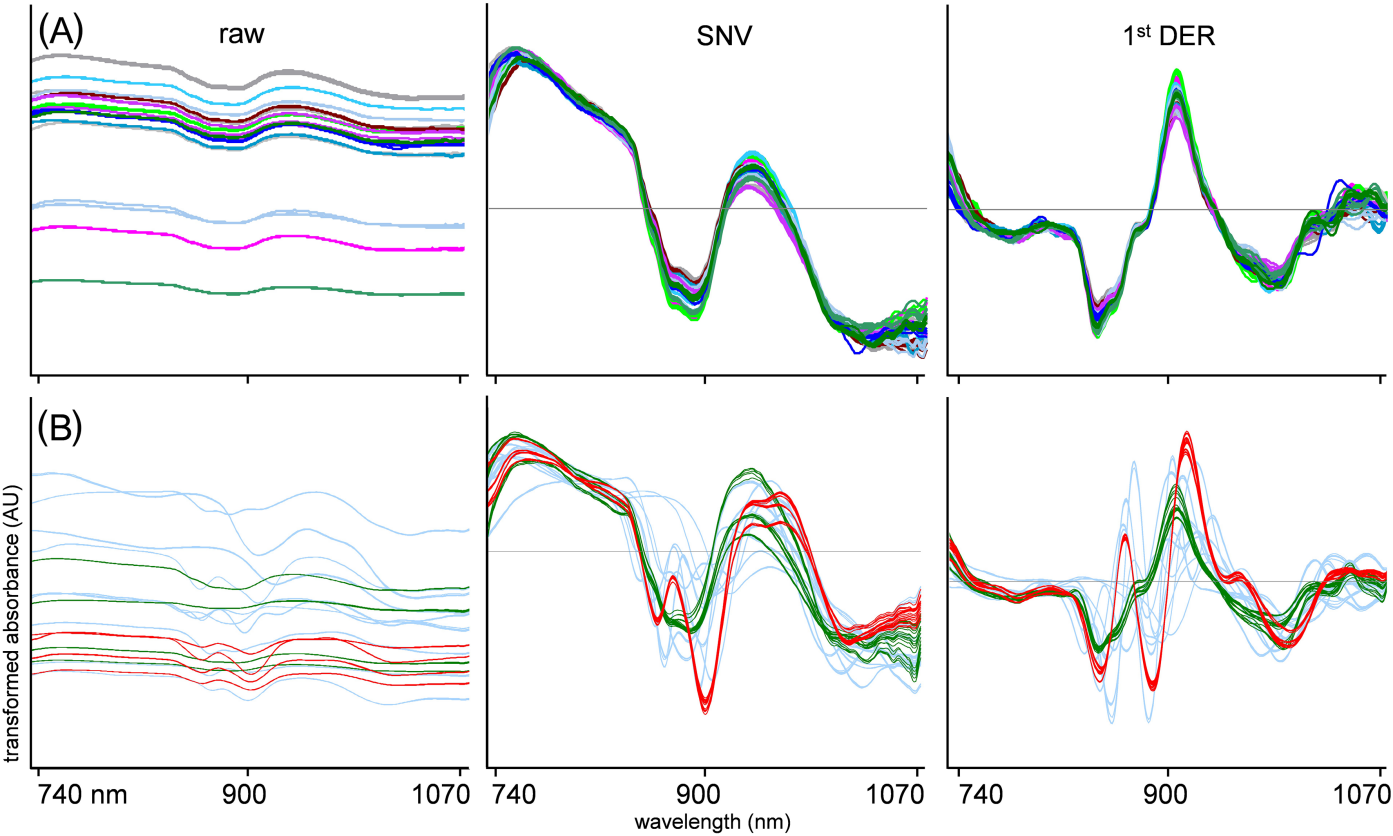
Effect of preprocessing on near‐infrared spectral data. A, Top‐row spectra are replicate scans of the same 86.6% cocaine HCl sample on 13 different scanners (5 spectra each). B, Bottom‐row spectra are scans from 4 cocaine HCl (green), 4 cocaine base (red), and 10 other common adulterants and other drugs (5 spectra each) measured using the same scanner. Spectra are shown in columns as raw spectral data (raw), after standard normal variate preprocessing (SNV) and SNV followed by Savitzky–Golay smoothing (19 datapoints) with a first‐order derivative (1st DER)

**FIGURE 3:**
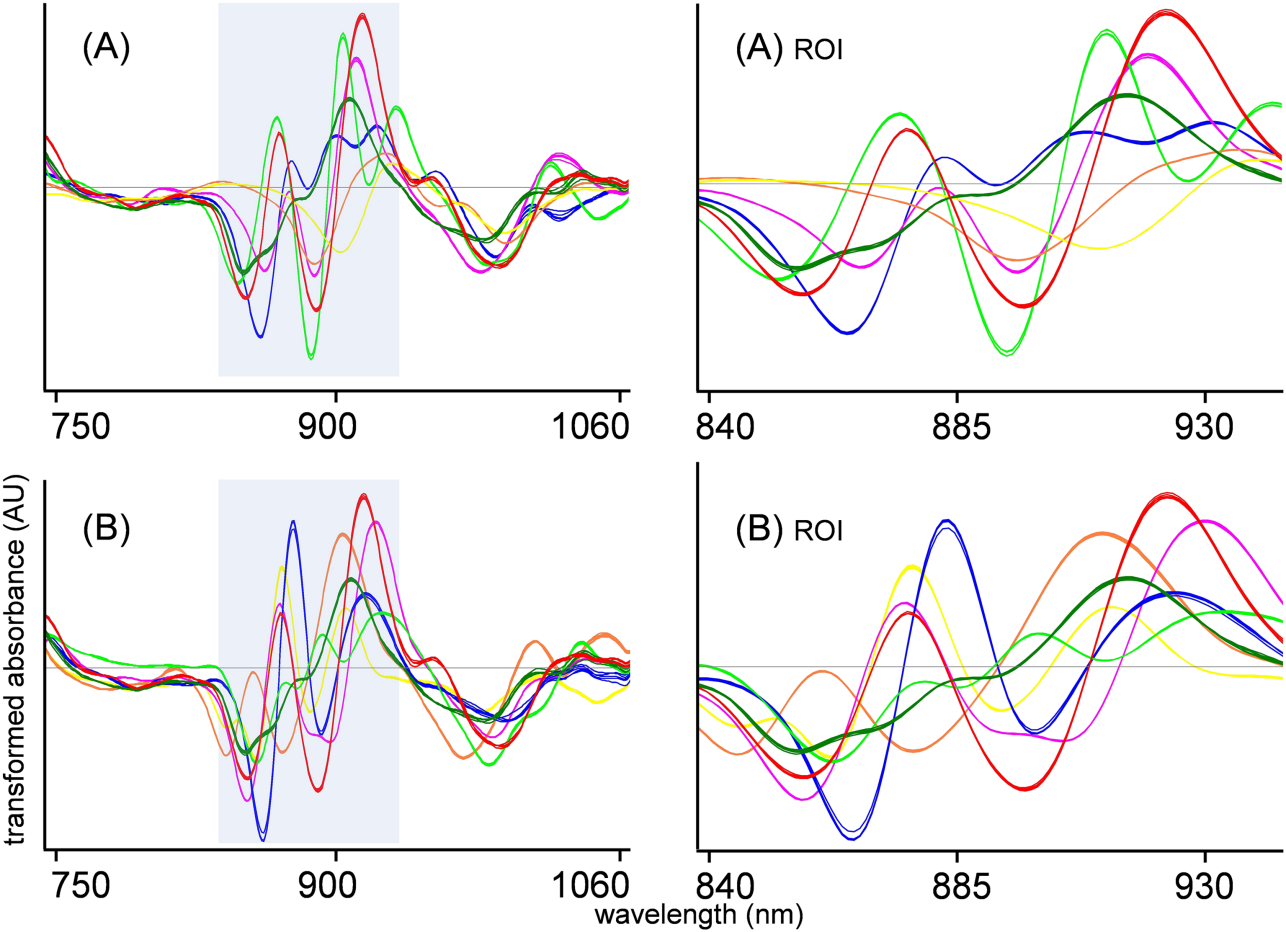
Near‐infrared spectra after standard normal variate–first derivative preprocessing of cocaine HCl (dark green) and cocaine base (red) in comparison with A, five common cutting agents and B, five common drugs, for both the full‐wavelength range and the 839–939 nm region of interest (ROI). Cutting agents (A): levamisole (blue), phenacetin (light green), lidocaine (pink), inositol (yellow), and mannitol (orange). Drugs (B): amphetamine (blue); methylenedioxymethamphetamine (light green), ketamine (pink), acetaminophen (yellow), and caffeine (orange)

For every individual sample within Set A and Set B, 65 replicate scans collected using 13 different scanners were available. In general, the five scans collected as replicates on a single scanner were visually similar; however, major intensity differences were observed among different scanners. The spectra marked as “raw” in Figure 2A show the unprocessed raw data from multiple scanners for an 86.6% cocaine HCl case sample. Each colored line indicates five replicate spectra from a single scanner, and each color indicates a single scanner. Additive baseline shifts could be clearly observed, with most scanners providing a near‐similar intensity, whereas three individual scanners returned a notably less‐intense signal. From observations of different samples, it was evident that this additive effect could not be attributed to individual poorly performing sensors, as sensors producing a low‐intensity signal for one sample did produce a high‐intensity signal for other samples. Two possible explanations for these additive effects are variation in sample vial positioning and signal scatter. As the glass vial containing the sample needs to be placed on top of the sensor before scanning (Figure 1), the variation in signal intensity might be due to the alignment of the sample. Operators were instructed to simply put the sample vial on top of the sensor such that both the NIR light source and the detector cell were covered by the vial. No special attention was given to the perfect alignment of the samples as this will also not be the case in the actual on‐scene analysis by police officers. However, with a vial diameter of 15 mm and a NIR light source and detector diameter 13 mm wide, there is limited tolerance. It is therefore possible that a vial placed more toward the detector surface will lose more light emitted from the sensor through the glass wall of the vial, thereby reflecting less signal. The other explanation given for the signal variation is the scattering effect of the material. Sample vials were necessarily touched and moved between analyses, and the powder in the vials was consequently shaken and redistributed. As the particle size of the powders might not be constant in actual case samples, more or less scattering resulting in varying signal intensities can occur. Sample scattering and the consequent signal variation is a regular phenomenon in diffuse reflectance NIR spectroscopy.^24^ Common strategies to correct for signal intensity and scattering effects are by means of data preprocessing. In our study, SNV processing proved to be a suitable technique for baseline correction (Figure 2A(ii)), in line with other studies.^22^, ^24^, ^30^ As NIR spectra, in general, and these small‐range spectra, in particular, are relatively information poor, a subsequent derivative preprocessing step is often suggested to put emphasis on the spectral differences. Consistent with an earlier NIR study on narcotics from Liu et al,^22^ a first‐order derivative following SNV was found sufficient for our data. A second‐order derivative, as suggested for cocaine analysis by Hespanhol et al,^23^ logically revealed even more spectral features on our data. For the differentiation of relatively pure substances, this preprocessing method could be the first choice due to the more‐prominent differences between compound spectra. However, the aim of this study was to correctly detect cocaine, even in complex mixtures of multiple compounds, and a second derivative could result in complex data sets in which the cocaine‐related signals are obscured, leading to poor model performance. Also, in cases of low NIR signal (ie, flat line reflection spectra), second derivative spectra can exhibit excessive noise. Figure 2 illustrates the effect of the preprocessing steps on our data, clearly showing reduced scanner‐to‐scanner variation and the additive baseline shift between scanners (A) and producing a reproducible and selective spectral signal for both cocaine HCl and cocaine base in comparison to other relevant substances (B). Cocaine HCl (snorting cocaine) and cocaine base (crack cocaine) produced a notably different NIR spectra in the 740–1070 nm range. This is consistent with an earlier work reporting different cocaine HCl and cocaine base NIR spectra in the 1000–1700 nm range, which can be easily explained by differences in IR vibrations between protonated cocaine in the salt form and the neutral cocaine molecule in the base form.^23^ Figure 3 shows the 740–1070 nm NIR spectra of protonated cocaine (cocaine HCl) and neutral cocaine (cocaine base), with a focus on spectral selectivity and emphasis on the individual spectra of the 10 most important other compounds encountered in casework. The same spectral information for the SNV‐second derivative spectra is shown in Figure S2 (supporting information). An overlay of the cocaine case samples with all other samples to visualize the selectivity in the 839–939 nm ROI area is shown in Figure S3 (supporting information). Because of these spectral differences, cocaine base and cocaine HCl are treated as different compounds in the detection model.

### 3.2 Model development

**FIGURE 4:**
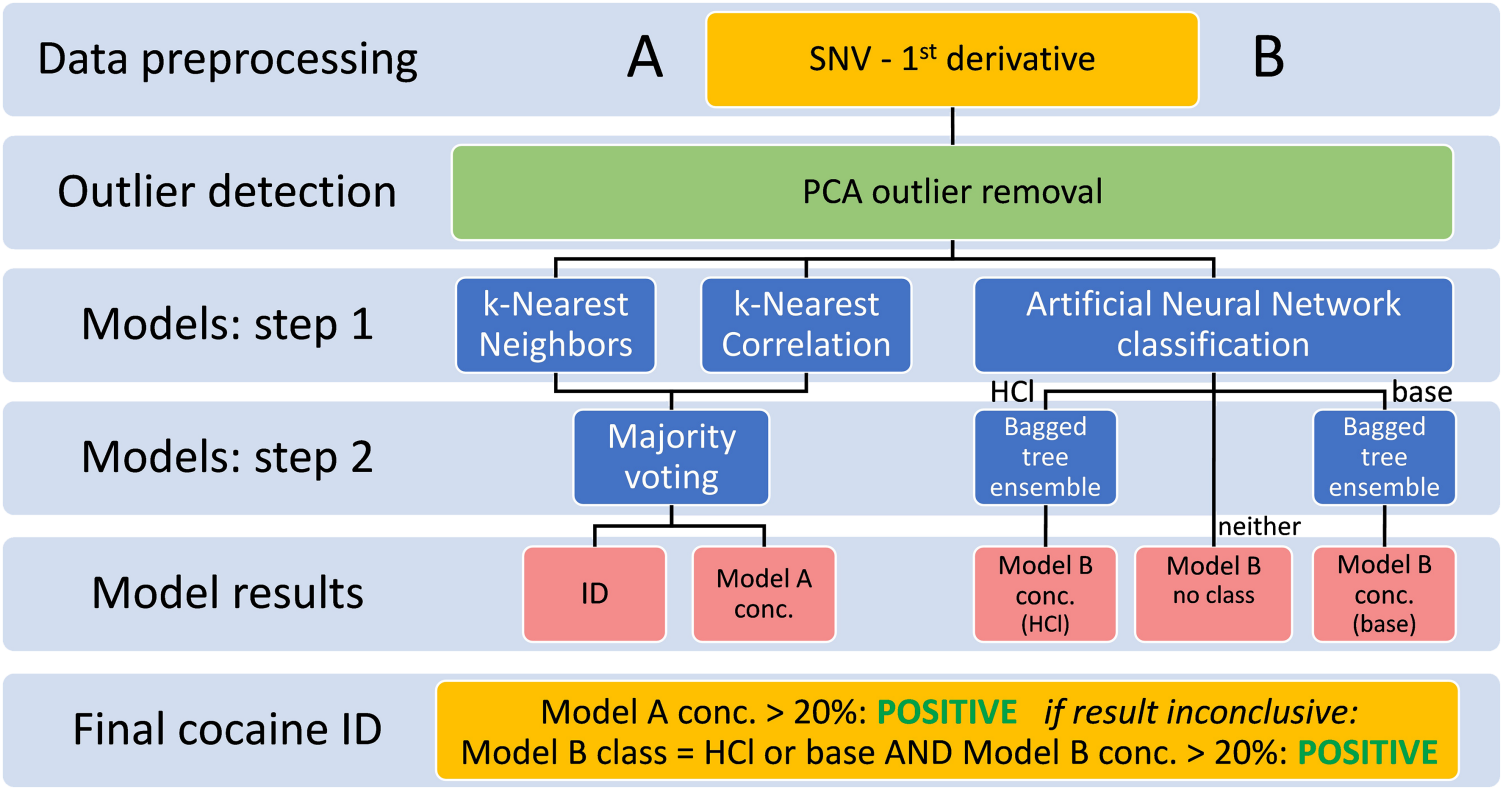
Schematic overview of the multistage cocaine identification model

A multistage model was developed with the aim of providing a robust and reliable NIR‐based solution for on‐scene detection of cocaine in powdered samples. The goal of this solution was to provide a reasonable suspicion to undertake judicial steps such as the subsequent seizure of materials and confirmatory analysis in a laboratory. Therefore, this method was targeted to provide minimal FP and FN results in representative actual case samples. In addition to the main model result being “positive” or “negative” for cocaine, prediction of the cocaine quantity and overall sample identity were produced by the model. A schematic representation of the developed model is given in Figure 4. As described in Section 3.1, the preprocessing step consisted of SNV followed by a first derivative. For model performance evaluation (Section 3.3), a second derivative and/or ROI selection were also applied. The k‐nearest neighbors models were cross‐validated on a “leave‐one‐sample‐out” basis—up to 65 scans of one sample were removed from the training set. For the ANN and bagged tree models, the data set was divided into 10 cross‐validation segments such that all individual replicate spectra from a single sample were left out in the same segment. This prevented the model from producing very optimistic results due to obvious similarities among replicates.^39^ After data‐preprocessing and cross‐validation segment creation, two separate models were used on the data.

#### 3.2.1 kNN submodels

**FIGURE 5:**
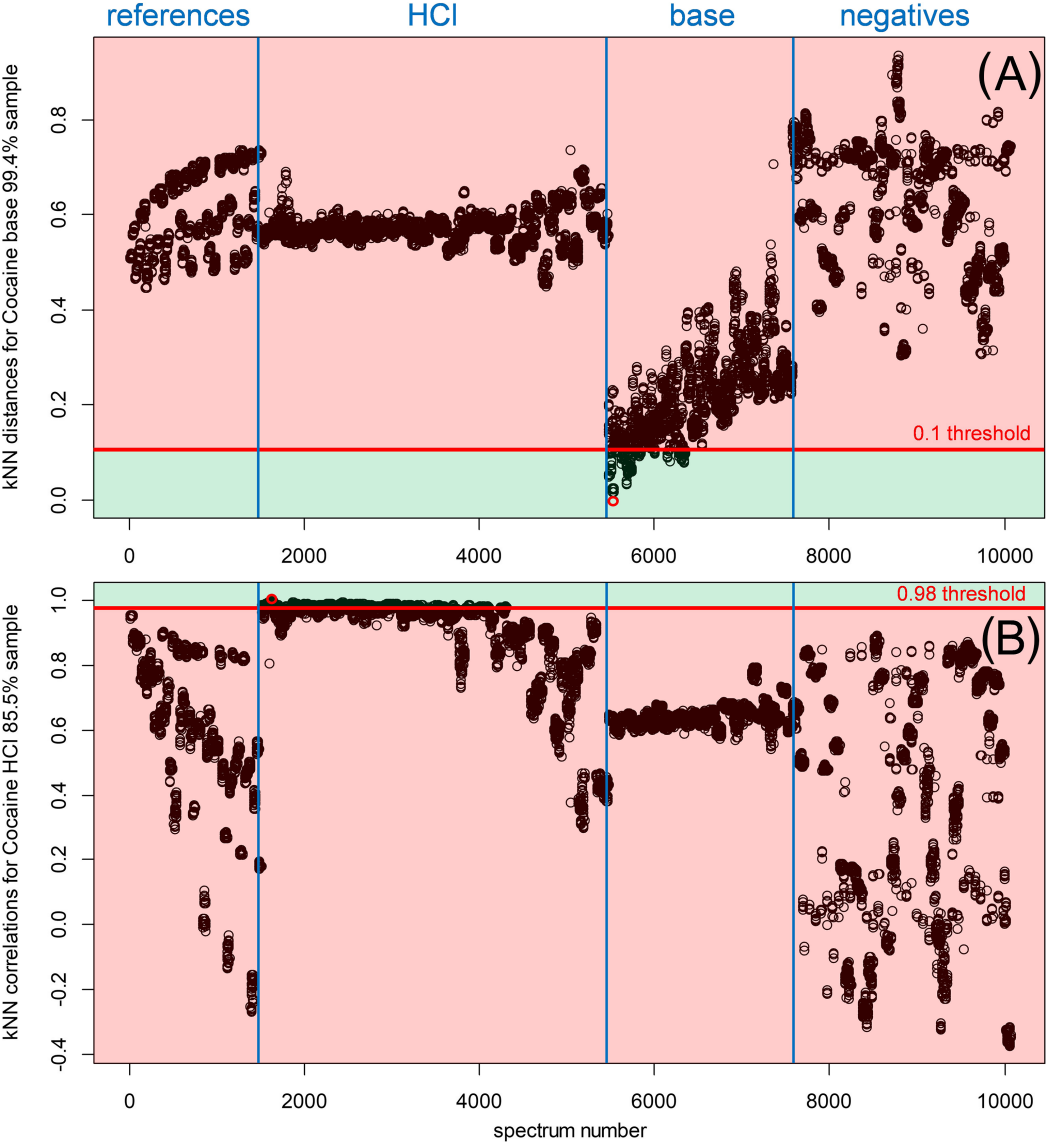
A, k‐nearest neighbor Euclidean distances plot of a cocaine base sample and B, Pearson correlation plot of a cocaine HCl sample, both plotted against all 10,059 model spectra. Spectra 1–1503 are from the diluted cocaine HCl reference set; spectra 1504–5465 are cocaine HCl case samples; spectra 5466–7639 are cocaine base case samples; spectra 7640–10,059 are various cutting agents, adulterants, and other drugs. All spectra are sorted by cocaine concentration within each group. The respective spectrum itself is marked in red. Model results were determined without this and all replicate spectra

The first submodel (depicted as A in Figure 4) is a dual‐stage kNN model. Its first step involves a PCA outlier removal, where individual scans were marked as outliers when the average Euclidean distance of a scan versus all other scans for the same sample is larger than the 99% quantile for that sample, based on the principal component (PC) scores 1–3 of a PCA on the total data set. In this way, 431 of 10,059 spectra were marked as outliers after SNV and first derivative preprocessing. As a next step, Euclidean distances and Pearson correlations were calculated for all combinations of individual spectra in the training sets (leave‐one‐sample‐out cross‐validation). For each individual spectrum, nine spectra with the smallest Euclidean distance (ie, NNs) and nine spectra with the highest correlation value (ie, nearest correlations) to the respective spectrum were considered. To reduce the possibility of an incorrect identification of an unknown spectrum (ie, a novel compound or mixture not yet included in the model), threshold values were applied. Thresholds of a maximal 0.1 distance and minimal 0.98 correlation proved satisfactory. Figure 5A shows an example of the Euclidean distances of all spectra toward a single cocaine base spectrum. It can be observed that only cocaine base class spectra exhibit clear similarities with the reference spectrum as indicated by a small distance or high correlation score. Also, a diagonal trend is visible within the cocaine base spectral group which is related to the cocaine content in the samples, which decreases from left to right as the samples are ordered as a function of concentration. In a similar fashion, a “match” with the relatively high‐concentration cocaine HCl spectra can be observed from the kNN Pearson correlations in the example in Figure 5B. For this figure the diagonal trend is absent because the cocaine HCl samples in the set typically are quite pure. Additional examples of kNN distances for both high‐, medium‐, and low‐percentage cocaine HCl and base spectra are shown in Figures S4 and S5 (supporting information). Examples of kNN correlation plots for the same spectra are given in Figures S6 and S7 (supporting information). kNN distances plots for the common cutting agents—levamisole, lidocaine, and acetaminophen—as well as the common drugs with a white powdery appearance—ketamine, amphetamine, mephedrone, MDMA, and methamphetamine—are shown in Figures S8 and S9 (supporting information). The red line in all kNN plots shows the threshold value of 0.1 for the Euclidean distance, and the dots in the green area are “matching” spectra that were further assessed in the next step. For all non‐cocaine spectra in Figures S8 and S9 (supporting information), it is evident that all spectra clearly exceed this threshold value. In Figure S8‐C (supporting information), some scans, originating from acetaminophen samples with low (6%–17%) cocaine content from the reference set, yield Euclidean distances between 0.1 and 0.2 close to the threshold of 0.1, which can be explained by the high acetaminophen content in these samples.

**FIGURE 6:**
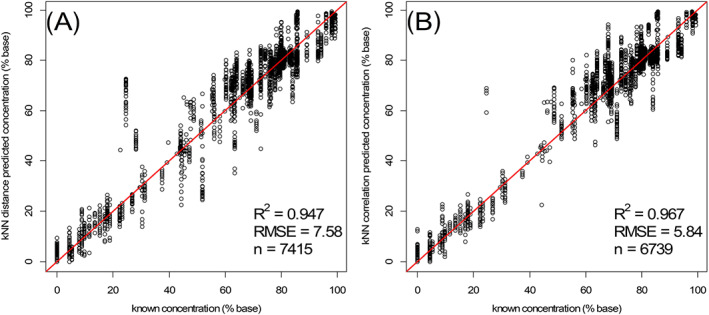
Known versus predicted cocaine concentrations from the A, k‐nearest neighbor (kNN) distances submodel and B, the kNN correlation submodel after SNV‐first derivative preprocessing of the 740–1050 nm spectral data

For each spectrum an identity (ID) was predicted from a majority voting of the known identities of the (at maximum) 18 NNs from both kNN models within the threshold values chosen. Next to the ID, a cocaine percentage was predicted for every individual spectrum out of the known cocaine concentrations of the NNs (Model A concentration in Figure 4‐A). This predicted concentration was the average of all selected NNs. In the situation where no NNs were present due to threshold limits, this model did not return a result. From both the kNN distances model and the kNN correlation model, the average predicted concentration plotted against the known concentration for all spectra (Figure 6) showed a clear linear trend with *R* ^2^ of 0.947 (kNN distances) and 0.967 (kNN correlations) and root‐mean‐square error (RMSE) values 7.58 and 5.84, respectively. It should be noted that for 9% of the cocaine case sample spectra and 62% of the spectra from the negative set, no result was available from this kNN submodel due to the threshold constraints. The latter high percentage for the negative samples is explainable as all 65 replicate spectra from the same compound were excluded during cross‐validation and *near neighbors* were identified only for some closely correlated mixtures or sample compositions that were included in both the reference and negative sets. This number of 'not available' (NA) results will decrease after maturation of the model and addition of more spectra to the database, as can already be observed from the results of the unknown case samples under Section 3.4.

#### 3.2.2 ANN—dual bagged tree regression submodels

The second model (depicted as B in Figure 4) is an ANN model with PCA pre‐scaling for classification between cocaine HCl and cocaine base classes followed by two separate bagged tree ensembles for predicting a concentration for spectra within each class. The ANN model is applied on the PCs following PCA data reduction of the spectral data such that 99% of the variation is included in the PCs. In this data set 19 PCs were used to satisfy this criterion. As a result, the ANN model predicts the salt form (HCl or base) in the form of a probability value for all spectra in the database. Spectra with probabilities within the 0–0.05 and 0.95–1 ranges are, respectively, assigned to the “HCl” or “base” class, whereas all spectra with probabilities outside these ranges are not assigned to a class. As a next step, two separate treebag regression models are trained for both cocaine types. A treebag, bagged tree, or bagging classification and regression trees is an ensemble of decision trees using a bootstrap aggregating (bagging) algorithm for regression or classification.^38^ These submodels were trained on all known cocaine HCl and cocaine base spectra, excluding outliers and performing cross‐validation in a similar fashion as the first model. Cocaine percentages are subsequently predicted for all individual spectra in the database.

#### 3.2.3 Final model decision on spectral and sample levels

**FIGURE 7:**
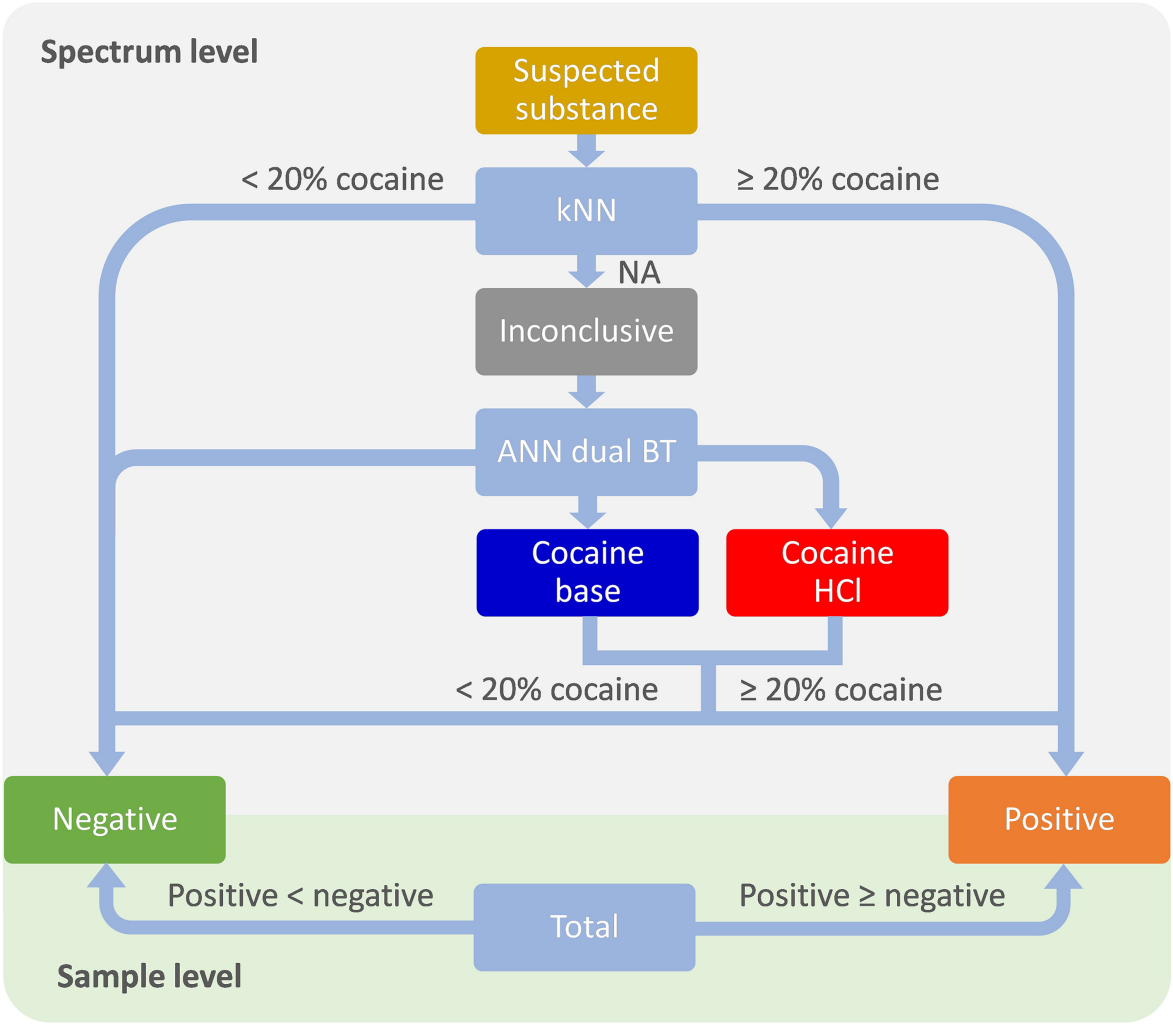
Model decision flowchart on the individual spectral level and sample level

For each spectrum the final model result is calculated as shown in Figure 7: when the average predicted cocaine concentration from the kNN submodel is above 20%, the final result is labeled positive, whereas when the predicted cocaine concentration is below 20%, the final result is negative. In both these cases, the result from the second submodel is ignored. When the kNN submodel was inconclusive, the result was determined by the second ANN–dual bagged tree submodel. In this case, the final result was positive when the predicted class for a spectrum was either “HCl” or “base” and the predicted concentration was above 20%. In all other cases, the result was negative. The reason for this submodel hierarchy is the better overall performance of the kNN submodel over the ANN–dual bagged tree submodel, as discussed in detail in Section 3.3. On the sample level, the final decision is made by majority voting of the results from all replicate spectra. Next to a final cocaine positive/negative decision, a sample ID is predicted based on the most similar spectra in the training data set.

### 3.3 Model performance evaluation

Table 1A and B presents the performance of the model, both on the individual spectrum level and on the sample level. The best performance for cocaine detection is achieved after SNV followed by second derivative preprocessing on the cocaine‐selective ROI part of the NIR spectrum. In this way, only 1.4% FP and FN results are encountered in individual spectra. On the sample level (thus applying a majority voting for replicate spectra), no FP results and 3.9% FN results are obtained, the latter originating from case samples with an actual cocaine content between 19% and 25%. When applying the NIR‐based model on samples with the only goal to detect or exclude cocaine, this is the suggested method of choice. However, in many forensic fields, additional information such as the identity of a “cocaine‐negative” sample is beneficial for on‐scene intelligence when no other indicative tests are available. Using only the cocaine‐selective part of the spectrum and thus excluding other spectral ranges, the potential to suggest an identity for a non‐cocaine unknown, such as another drug compound by the kNN models, is minimized. For predicting unknown samples on the model, the use of the full 740–1070 nm spectrum after SNV‐first derivative is therefore suggested. The most critical parameter in a forensic test often is the FP rate, as a FP result might lead to wrong judicial action. On the sample level, the only FP observed was for the cutting agent diltiazem after SNV‐first derivative‐ROI preprocessing. For this compound 36 spectra were predicted as cocaine positive, whereas 30 spectra were predicted as cocaine negative. Most FP matches for diltiazem corresponded with kNN‐model matches with a 45% cocaine HCl: paracetamol sample exploiting similar spectral features in the ROI as diltiazem. This indicates the selectivity limits of the ROI‐based approach. When looking at the false negatives, a majority originated from sub‐30% cocaine references from Set REF, no actual case samples (Set A) gave a FN result for the SNV‐second derivative data, and only one 53.5% cocaine base sample was labeled as negative after SNV‐first derivative data preprocessing. Individual FN spectra for case samples were in all other cases overruled by true‐positive (TP) results in the majority voting process. A general trend observed was that the ROI‐based approaches appeared to be more sensitive. This phenomenon can be explained by the increased focus on the major spectral features of cocaine, which increases the sensitivity with respect to cocaine detection but can also result in reduced selectivity and thus increased FP rates.

**TABLE 1:** Performance characteristics of the model decision flowchart as indicated in Figure 4 for the various forms of preprocessing

Preprocessing	True positives	False negatives	True negatives	False positives
*A* *Model performance on individual scans (9459 scans)*				
SNV‐first derivative	97.2% (6632)	2.8% (189)	98.2% (2591)	1.8% (47)
SNV‐second derivative	97.1% (6625)	2.9% (196)	98.3% (2594)	1.7% (44)
SNV‐first derivative‐ROI	97.8% (6668)	2.2% (153)	97.5% (2571)	2.5% (67)
SNV‐second derivative‐ROI	98.6% (6727)	1.4% (94)	98.6% (2600)	1.4% (38)
*B* *Model performance on sample level (316 samples)*				
SNV‐first derivative	92.6% (214)	7.4% (17)	100% (85)	0% (0)
SNV‐second derivative	87.9% (203)	12.1% (28)	100% (85)	0% (0)
SNV‐first derivative‐ROI	96.5% (223)	3.5% (8)	98.8% (84)	1.2% (1)
SNV‐second derivative‐ROI	96.1% (222)	3.9% (9)	100% (85)	0% (0)
*C External validation by unknown case material (625 scans)*				
SNV‐first derivative	90.6% (281)	9.4% (29)	95.6% (301)	4.4% (14)
SNV‐second derivative	92.3% (286)	7.7% (24)	94.6% (298)	5.4% (17)
SNV‐first derivative‐ROI	93.5% (290)	6.5% (20)	91.7% (289)	8.3% (26)
SNV‐second derivative‐ROI	96.5% (299)	3.5% (11)	95.2% (300)	4.8% (15)

*Notes.* A and B are cross‐validation results based on all case samples, negative samples, and all cocaine reference samples above 20%. C shows results from external validation samples not used in model development.

Abbreviations: ROI, region of interest; SNV, standard normal variate.

Table 2 presents the performance of the submodels that when combined provide the final model results. Additional details are presented in Table S1 (supporting information). In general, the kNN submodel gives the best performance with greater than 99% average accuracy. The limitation of this model is the number of inconclusive results when no *near neighbors* are identified within the thresholds. For the case samples, this is true for only 9% of the entire sample set, whereas more than half of the negatives do not give a match. As described earlier, the latter is an expected result due to the “leave all from the same sample out” cross‐validation. This problem will likely disappear when more negative samples are included in the model; however, novel substances will most probably yield an inconclusive result for this submodel. The second submodel, the ANN–dual tree bagging model, yields an average overall correct assessment in 95% of all samples considered, but contrary to the first submodel, a classification is obtained for all spectra. As the final model conclusion gives priority to the result from the more‐reliable kNN submodel, the result from the second submodel must be considered only for those spectra for which no result from the first submodel was obtained. These values are given in the bottom part of Table 2. A notable result is the 0% TP after SNV‐first derivative‐ROI preprocessing. This can be explained by the high performance of the first kNN submodel, already predicting 6743 of 6821 spectra for positive samples, thus leaving only the 78 most difficult samples for the next model. For the other preprocessing variants, the number of spectra assessed by the ANN model was six to eight times higher.

**TABLE 2:** Performance characteristics of the separate submodels

Preprocessing	True positives (%)	False negatives (%)	True negatives (%)	False positives (%)	# pos	# neg
*k‐Nearest neighbors (submodel A) performance*						
SNV‐first derivative	98.9	1.1	99.3	0.7	6184	1011
SNV‐second derivative	98.2	1.8	100	0.0	6277	1197
SNV‐first derivative–ROI	98.9	1.1	96.6	3.4	6743	1543
SNV‐second derivative‐ROI	99.4	0.6	99.7	0.3	6377	1188
*ANN‐dual tree bagging (submodel B) performance*						
SNV‐first derivative	96.1	3.9	97.3	2.7	6821	2638
SNV‐second derivative	88.9	11.1	98.0	2.0	6821	2638
SNV‐first derivative–ROI	89.4	10.6	96.6	3.4	6821	2638
SNV‐ second derivative‐ROI	95.1	4.9	98.6	1.4	6821	2638
*ANN‐dual tree bagging (submodel B) performance on submodel A inconclusive spectra*						
SNV‐first derivative	81.0	19.0	97.5	2.5	637	1627
SNV‐second derivative	85.3	14.7	96.9	3.1	544	1441
SNV‐first derivative–ROI	0.0	100	98.6	1.4	78	1095
SNV‐second derivative‐ROI	87.6	12.4	97.7	2.3	444	1450

*Notes.* Percentages are based on all spectra from case samples, negative samples, and reference samples with concentration above 20%, being 9459 spectra. The performance of submodel 2 is shown for comparison as this submodel is applied only on the inconclusive spectra following submodel 1.

Abbreviations: ANN, artificial neural network; ROI, region of interest; SNV, standard normal variate; # pos, number of positives; # neg, number of negatives.

**FIGURE 8:**
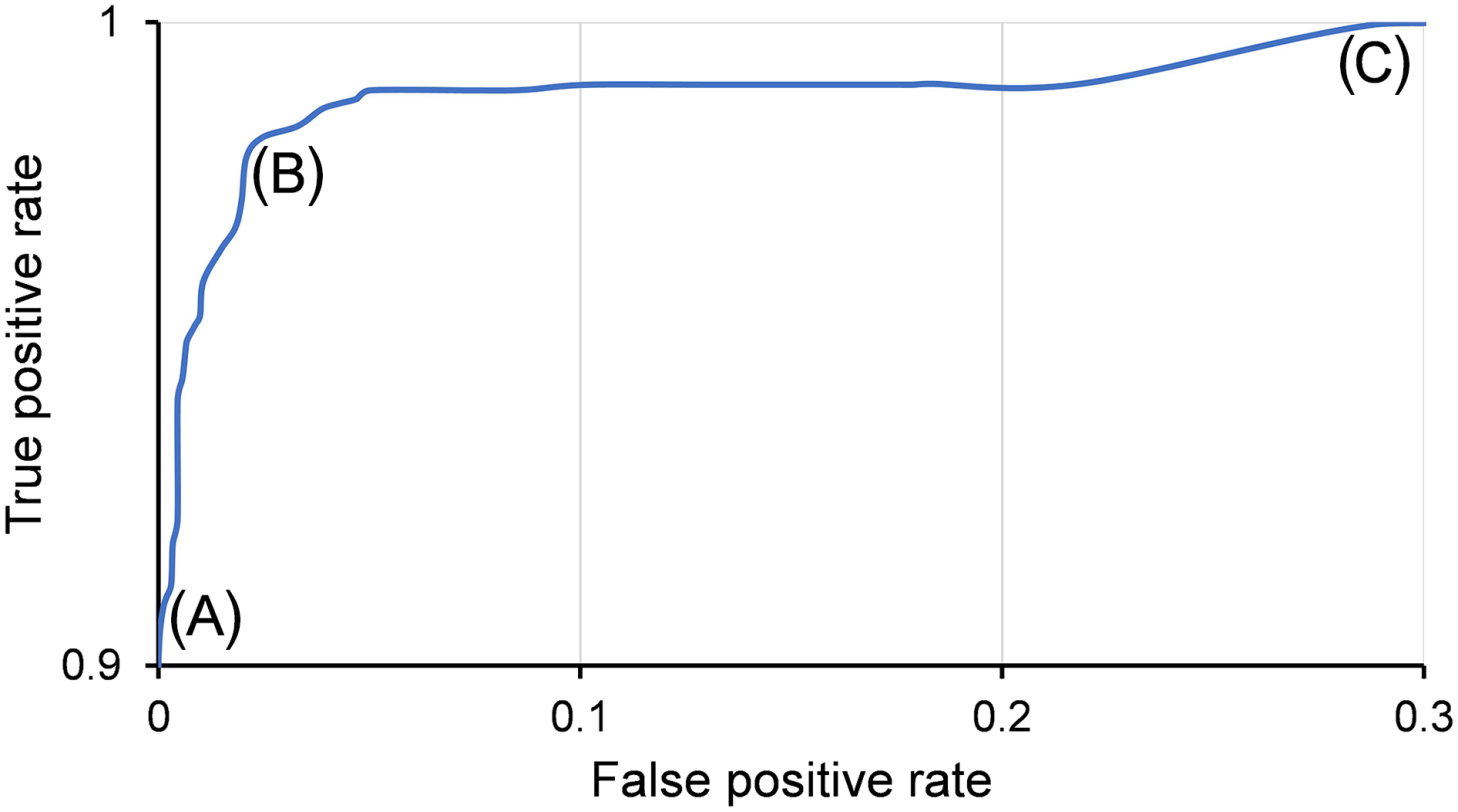
True‐positive versus false‐positive rate plot of the near‐infrared‐based model on all Set A case samples, all Set B negatives, and references between 18.8 and 100 wt% cocaine. A, The 0% false‐positive threshold observed at 36 wt% cocaine; B, the optimal accuracy at 18 wt% cocaine, and C, the <0.1% false‐negative threshold at 2 wt% cocaine

It is important to emphasize that the FP versus FN rate of the model could be adjusted by varying the weight percentage–cocaine threshold. In this way, the optimal model performance can be “tuned” for specific forensic or security use. From a legal perspective, all cocaine‐containing samples regardless of concentration are considered controlled materials in the Netherlands. A negligible FN rate could be selected by using a low weight percentage–cocaine threshold, but this comes at the price of a higher FP rate. In this case, more samples are (incorrectly) sent to the laboratory for confirmatory analysis. This results in wasted laboratory capacity and possibly wrong arrests and pre‐detention. On the contrary, in situations where FNs are manageable but FPs are unwanted (eg, situations where other indicative on‐site tests are also available but where no subsequent legal action or laboratory analysis is performed), a higher weight percentage threshold could be selected. The performance of the overall optimal first derivative model on the full spectra was evaluated by determining the TP and FP rates of the model at various weight percentage–cocaine thresholds. Figure 8 shows the FP versus TP curve for a set of 9459 spectra, including all case sample and negative spectra. From these data various optimal threshold values could be derived depending on the specific forensic requirements. Point A in Figure 8 marks the 36 wt% threshold level where no FPs were observed, but this comes at a cost of 9.9% FNs. Point B in Figure 8 shows the optimal accuracy around an 18 wt% cocaine cutoff, and point C in Figure 8 gives the optimum for minimal (<0.1%) FNs that corresponds 28.8% FPs. This optimum was observed at a 2 wt% cocaine‐cutoff threshold. These characteristics clearly show that the threshold could be used to optimize the model for specific forensic settings in which certain FP or FN percentages could be acceptable. In this study, a generic 20% threshold was applied. This was found suitable for the actual Dutch cocaine market where sub‐20% cocaine content case samples are rarely encountered and the reported average cocaine content is above 50 wt%.^2^, ^3^, ^4^, ^5^ Although the observed FP and TP rates were satisfactory for an indicative testing method, it must be noted that applying such a threshold will deliberately introduce FN results for samples with cocaine content below this threshold. Although it is acceptable in specific situations such as indicative testing, narcotic legislation in many countries does not contain concentration limits for controlled substances, and even low‐concentrated samples are thus illicit.

### 3.4 External validation and unknown samples

As a first demonstration for the applicability of the developed model, 54 seized off‐white case samples were randomly selected by the Amsterdam Police Laboratory staff between October 2019 and March 2020. In addition, 22 seized case samples known to contain different drug types, mainly NPSs, were selected. Furthermore, external validation samples were prepared by diluting a pure cocaine HCl sample with levamisole, inositol, and caffeine from 0% to 100% with 10% intervals. All these samples were scanned fivefold, and spectral data were processed and predicted using the developed model. The original laboratory results from GC–MS, FT‐IR, and Raman analyses were used for comparison. The results on the individual scan level, presented in Table 1C, are consistent with the model performance from the cross‐validation. Of the 54 case samples, 34 were reported as “cocaine containing” by the laboratory (based on GC–MS results). Within these, 31 were correctly predicted as cocaine positive using the NIR‐based model. The three FN samples contained an unusual low level of cocaine (estimated between 5% and 17%) below the threshold as applied in the model. A possible explanation of this relatively high number of low level cocaine samples might be some selection bias from the forensic experts selecting more “out‐of‐the‐ordinary” case samples for this study. The other 20 case samples were found to contain no illicit compound or contain another drug from prior laboratory data. Within this group, only one sample produced an FP result for cocaine on the NIR model. The GC–MS data revealed that this specific specimen contained amphetamine adulterated with caffeine. As the first kNN submodel was inconclusive for this sample, the FP result originated from the ANN submodel. The NPS and other drug‐containing case samples produced no FP results for cocaine, and both cocaine HCl and cocaine base samples were correctly identified. In most cases, no identity was predicted by the kNN submodel, which was correct as these novel substances were not included in the database. Euclidean distances and Pearson correlations fell outside the threshold limits for all database spectra as shown for a synthetic drug (2C‐B) containing sample in Figure S10. When the model did predict an identity, it was in most cases consistent with the GC–MS results. Some exceptions were two ketamine‐containing samples misidentified as a levamisole:phenacetin mixture, and a 3‐methylmethcathinone‐containing sample was misidentified as containing 4‐methylmethcathinone (mephedrone). The complete results for all case samples are shown in Tables S2 and S3 (supporting information).

**FIGURE 9:**
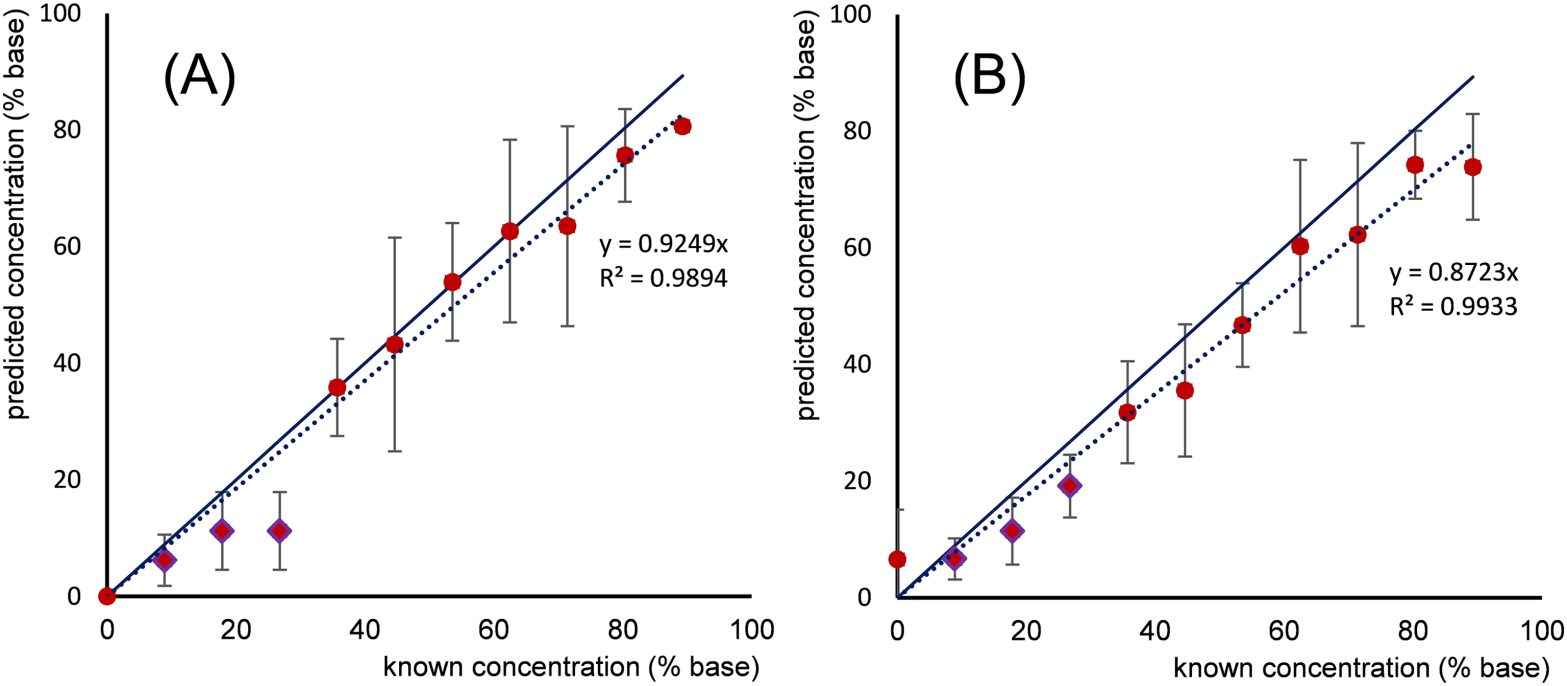
Predicted versus actual concentrations of external validation samples consisting of binary 0%–100% cocaine HCl mixtures with levamisole, caffeine, and inositol, each scanned fivefold. A, Results after standard normal variate (SNV)‐first derivative‐full spectrum preprocessing; B, after SNV‐second derivative‐region of interest preprocessing. Diamond‐shaped datapoints originate from the ANN‐treebag regression model; all other datapoints originate from the k‐nearest neighbor model. The solid blue line shows perfect prediction; the dotted blue line shows the curve derived from the actual data

For the external validation samples, all samples with an actual cocaine content of 40% and above were predicted as cocaine positive by the model, with the exception of one sample containing 40% cocaine in levamisole. For this sample, 2 out of 5 spectra were predicted to be positive and 3 out of 5 were negative , thus giving an overall negative result in the majority voting. It must be emphasized that all samples with a predicted cocaine content between 0% and 20% are deliberately labeled as negative in the model when the default 20% cutoff threshold is applied. Many samples with an actual concentration between 20% and 30% were predicted to contain less than 20% cocaine, thus giving a negative model result. It should be noted that the quantification of cocaine content is not the goal of this approach, and predicted concentrations are used only for a check against the threshold. However, the quantitative performance of the technique was further assessed to provide insight into its robustness and potential future applications. As shown in Figure 9A, a good correlation is achieved with a notable exception for the 10%–30% cocaine‐content samples. This is explained by the priorities in the model design where the kNN model is dominant over the other model. For these 10%–30% samples, the kNN model was inconclusive, and the concentration was thus predicted by the ANN‐treebag regression model. When the standard preprocessing was used on full spectral data, this latter model was more conservative. Considering ROI selection (Figure 9B), a better correlation at the lower concentrations is observed due to the increased focus on the 839–914 nm cocaine spectral signals. This is consistent with earlier results (Section 3.3) but has the disadvantage of limited selectivity for the other compounds.

### 3.5 Discussion

This is the first study to demonstrate cocaine detection using NIR spectral data covering a limited‐wavelength range and using a low‐cost handheld device. Although the first results show impressive performance, optimization of several aspects might even further increase the performance of the model and reduce the risk of erroneous results. In this study limited attention is given to the threshold and cutoff values that determine the preferred routes within the model. For example, stricter kNN thresholds will force more spectra to be predicted by the ANN model, and the optimal number of NNs could be different for more uncommon spectra. For practical implementation, the reference database must be expanded with a large variety of cocaine samples to create a more robust model. One limitation of the machine learning model is that it does not exclusively focus on spectral features originating from the illicit compound itself (ie, cocaine in this case). This is contrary to traditional IR, Raman, or mass spectral library search approaches where database spectra originate only from (and could even be theoretically explained as belonging to) the reference compound. Cocaine samples can vary widely in composition,^40^ and a possible risk is that complex mixtures produce FP results as the overall spectra exhibit random and unexpected similarities to TP reference samples. One way to reduce this risk is to train the model on many different samples. As subsequent GC–MS analysis is used routinely in a forensic laboratory, new spectra could simply be added to the model database by updating the metadata of the original spectra with the GC–MS results. This way, the database could be updated almost in real time, and the model performance would be automatically adapted for changing compositions of street samples over time. For example, when cocaine mixed with a novel cutting agent is encountered for the first time, the model might produce a (false) negative result. However, after adding the spectra to the database, a new sample having the same composition will “match” with the first one as a near‐neighbor. In this way, the model will rapidly adapt to changes in the cocaine market.

Other possible improvements are the analysis through various types of packaging materials. In this model, all spectra were collected through glass vials, which in a practical setting still means that the officer conducting the NIR measurements needs to transfer the suspect material from its original packaging to a suitable glass container. Earlier studies report on the limited effect of plastic packaging on the NIR signal.^41^, ^42^ Future studies on the effect of packaging materials might therefore be beneficial as this will even further reduce the risk of the investigating officer being exposed to harmful substances. Also, in this study only white‐ or off‐white‐colored powders were included in the model as this is the most common appearance of cocaine samples. The applicability of the model on more intensely colored samples is thus unknown. Additional experiments on the influence of colored substances on the NIR spectrum could provide valuable insight into the robustness of the model. Another interesting outlook is data fusion with other types of spectral data from handheld equipment. In this way, a combination of complementary techniques that are now used only for indicative testing might for some sample types produce sufficient evidence to eliminate the need of laboratory analysis for final confirmation. In such an ideal situation, an on‐scene analysis in seconds would suffice to present the required evidence in court.

## 4 CONCLUSION

Low‐cost, handheld NIR scanners using a small 740–1070 nm wavelength range provide sufficient spectral selectivity for robust cocaine detection in illicit‐drug suspected case samples. The confined spectral features were exploited by SNV‐first derivative preprocessing followed by a multistage model consisting of various machine learning algorithms. A model database was constructed using 10,059 NIR spectra, recorded with 15 different handheld NIR devices using a set of 180 samples. To achieve a robust and representative model, recent case samples with known identity and concentration as well as a large set of known bulking agents, adulterants, and other drugs were incorporated in the data set. The first and most accurate stage in the model was a kNN Euclidean distances and Pearson correlation algorithm. This model predicted cocaine concentration for unknown spectra by finding the nearest neighbor spectra in the database. In this way, 98.9% true‐positive and 99.3% true‐negative (TN) accuracy was achieved. By using model thresholds, novel spectra not yet recognized by the model were labeled as inconclusive. In the next step, an ANN predicted the cocaine type (ie*,* base or HCl), whereas bagging tree regression models predicted the cocaine content for all resulting spectra. In the final decision criteria, a cutoff threshold could be used to reduce FP results at the cost of increased FN results for low level cocaine samples. As the occurrence of this type of samples in actual casework is very low, this is acceptable for an on‐scene indicative test. FP versus TP plots provide insight into the model performance and could be used to determine a cutoff threshold suitable for the specific situation. The overall model yielded TP and TN rates of 97.2% and 98.2%, respectively, at a 20 wt% cocaine cutoff threshold. The external validation on 54 recent case samples and 33 diluted cocaine standards demonstrated practical applicability. This NIR‐based approach provides a robust, rapid, and intrinsically saver procedure as minimal handling of potentially hazardous substances is required. For future use of the model, unknown spectra could easily be added to the database by updating the metadata with the confirmed identity from validated lab results. In this way, an intelligent model that can rapidly adapt to the ever‐changing cocaine market is established. Validated laboratory data ensure optimal performance of the model at any given time as police officers scan suspect samples using handheld NIR devices. This model is accessed centrally as the NIR spectral data are sent to a cloud environment using Bluetooth and WiFi/4G connection of the smartphone of the officer. Thus, forensic drug analysis experts can safeguard the quality of the on‐scene analysis “from a distance” and in “real time” as the model returns an analysis outcome to the smartphone almost instantly.

## CONFLICT OF INTEREST

The authors declare no competing interest.

## AUTHOR CONTRIBUTIONS

Ruben F. Kranenburg: methodology, investigation, formal analysis, software, data curation, and writing—original draft. Joshka Verduin: investigation, formal analysis, visualization, and writing—original draft. Yannick Weesepoel: methodology, investigation, data curation, and writing—review and editing. Martin Alewijn: software, data curation, and writing—review and editing. Marcel Heerschop: investigation and project administration. Ger Koomen: resources and supervision. Peter Keizers: investigation. Frank Bakker: investigation and writing—review and editing. Fionn Wallace: investigation. Annette van Esch: investigation. Annemieke Hulsbergen: investigation and writing—review and editing. Arian C. van Asten: conceptualization, investigation, methodology, supervision, and writing—review and editing.

## Supporting information


**Figure S1** Effect of smoothing during SNV‐first derivative pre‐processing for noise removal
**Figure S2** NIR spectra after SNV‐second derivative pre‐processing of cocaine HCl and cocaine base in comparison with 5 common cutting agents and 5 common drugs
**Figure S3** Overlay of NIR spectra from cocaine base samples; cocaine HCl samples; cutting agents and other compounds
**Figure S4** k‐Nearest Neighbors distances plots of a high, medium and low level cocaine HCl case sample on NIR spectra after SNV‐first derivative pre‐processing
**Figure S5** k‐Nearest Neighbors distances plots of a high and medium level cocaine base case sample on NIR spectra after SNV‐first derivative pre‐processing
**Figure S6** k‐Nearest Neighbors correlations plots of a high, medium and low level cocaine HCl case sample on NIR spectra after SNV‐first derivative pre‐processing
**Figure S7** k‐Nearest Neighbors correlations plots of a high and medium level cocaine base case sample on NIR spectra after SNV‐first derivative pre‐processing
**Figure S8** k‐Nearest Neighbors distances plots of levamisole, lidocaine, acetaminophen and ketamine on NIR spectra after SNV‐first derivative pre‐processing
**Figure S9** k‐Nearest Neighbors distances plots of amphetamine, mephedrone, MDMA powder and methamphetamine on NIR spectra after SNV‐first derivative pre‐processing
**Figure S10** kNN distances and correlations plots for a non‐matching unknown “2C‐B” sample projected on all database spectra
**Table S1** Absolute performance characteristics of the full model and parts of the model after various forms of pre‐processing
**Table S2** Results of unknown case samples projected on the model and compared with laboratory results
**Table S3** Results of various NPS and drug containing case samples projected on the model and compared with laboratory results
