# Supplementary material for: Rapid and robust on‐scene detection of cocaine in street samples using a handheld near‐infrared spectrometer and machine learning algorithms
Source: Drug Test Anal. 2020 Jul 27;12(10):1404–18. doi: 10.1002/dta.2895 (PMC7590077; doi:10.1002/dta.2895)
Supplement: Supplementary file 1 — Figure S1 Effect of smoothing during SNV‐first derivative pre‐processing for noise removal Figure S2 NIR spectra after SNV‐second derivative pre‐processing of cocaine HCl and cocaine base in comparison with 5 common cutting agents and 5 common drugs Figure S3 Overlay of NIR spectra from cocaine base samples; cocaine HCl samples; cutting agents and other compounds Figure S4 k‐Nearest Neighbors distances plots of a high, medium and low level cocaine HCl case sample on NIR spectra after SNV‐first derivative pre‐processing Figure S5 k‐Nearest Neighbors distances plots of a high and medium level cocaine base case sample on NIR spectra after SNV‐first derivative pre‐processing Figure S6 k‐Nearest Neighbors correlations plots of a high, medium and low level cocaine HCl case sample on NIR spectra after SNV‐first derivative pre‐processing Figure S7 k‐Nearest Neighbors correlations plots of a high and medium level cocaine base case sample on NIR spectra after SNV‐first derivative pre‐processing Figure S8 k‐Nearest Neighbors distances plots of levamisole, lidocaine, acetaminophen and ketamine on NIR spectra after SNV‐first derivative pre‐processing Figure S9 k‐Nearest Neighbors distances plots of amphetamine, mephedrone, MDMA powder and methamphetamine on NIR spectra after SNV‐first derivative pre‐processing Figure S10 kNN distances and correlations plots for a non‐matching unknown “2C‐B” sample projected on all database spectra Table S1 Absolute performance characteristics of the full model and parts of the model after various forms of pre‐processing Table S2 Results of unknown case samples projected on the model and compared with laboratory results Table S3 Results of various NPS and drug containing case samples projected on the model and compared with laboratory results [file DTA-12-1404-s001.docx]

**Supplemental Information**

for

**Rapid and Robust On-Scene Detection of Cocaine in Street Samples using a Handheld Near Infrared Spectrometer and Machine Learning Algorithms**

Ruben F. Kranenburg^1,2,*^, Joshka Verduin^1,2^, Yannick Weesepoel^3^, Martin Alewijn^3^, Marcel Heerschop^4^, Ger Koomen^4^, Peter Keizers^5^, Frank Bakker^5^, Fionn Wallace^6^, Annette van Esch^6^, Annemieke Hulsbergen^6^, Arian C. van Asten^2,7^

^1^ Dutch National Police, Unit Amsterdam, Forensic Laboratory, Kabelweg 25, Amsterdam 1014 BA, The Netherlands

^2^ Van ’t Hoff Institute for Molecular Sciences, University of Amsterdam, Postbus 94157, Amsterdam 1090 GD, The Netherlands

^3^ Wageningen Food Safety Research part of Wageningen University and Research, Akkermaalsbos 2, Wageningen 6708 WB, The Netherlands

^4^ Dutch Customs Laboratory, Kingsfordweg 1, Amsterdam 1043 GN, The Netherlands

^5^ National Institute of Public Health and the Environment (RIVM), Antonie van Leeuwenhoeklaan 9, Bilthoven 3721 MA, The Netherlands

^6^ Netherlands Forensic Institute (NFI), Laan van Ypenburg 6, Den Haag 2497 GB, The Netherlands

^7^ Co van Ledden Hulsebosch Center (CLHC), Amsterdam Center for Forensic Science and Medicine, Postbus 94157, Amsterdam 1090 GD, The Netherlands

^*^ Corresponding author. *E-mail address:* ruben.kranenburg@politie.nl (R.F. Kranenburg).

Fig. S1. Effect of smoothing during SNV-1^st^ derivative pre-processing for noise removal using windows of A) 3 datapoints; B) 7 datapoints; C) 11 datapoints; D) 15 datapoints; E) 19 datapoints and F) 23 datapoints. Red circles indicate the spectral areas where excessive noise is observed.

Figure S2. NIR spectra after SNV-2^nd^ derivative pre-processing of cocaine HCl (dark green) and cocaine base (red) in comparison with 5 common cutting agents (A) and 5 common drugs (B), both the full NIR spectrum and the 839 – 914 nm region of interest (ROI). Cutting agents (A): levamisole (blue); phenacetine (light green); lidocaine (pink); inositol (yellow); mannitol (orange). Drugs (B): amphetamine (blue); MDMA (light green); ketamine (pink); acetaminophen (yellow); caffeine (orange). Overlays of 5 scans per compound.

Figure S3. Overlay of NIR spectra from cocaine base (red, 39 samples, 1949 spectra, 35 – 99%); cocaine HCl (green, 48 samples, 3664 spectra, 19 – 86%); cutting agents and other compounds (light blue, 39 samples, 2370 spectra). Spectra after SNV – 1^st^ derivative (A) and SNV – 2^nd^ derivative (B) pre-processing.

**C**

**B**

**A**

Figure S4. k-Nearest Neighbors distances plots of a high (A), medium (B) and low (C) concentrated cocaine HCl case sample on NIR spectra after SNV-1^st^ derivative pre-processing.

**A**

**B**

Figure S5. k-Nearest Neighbors distances plots of a high (A) and medium (B) concentrated cocaine base case sample on NIR spectra after SNV-1^st^ derivative pre-processing.

**C**

**B**

**A**

Figure S6. k-Nearest Neighbors correlations plots of a high (A), medium (B) and low (C) concentrated cocaine HCl case sample on NIR spectra after SNV-1^st^ derivative pre-processing.

**B**

**A**

Figure S7. k-Nearest Neighbors correlations plots of a high (A) and medium (B) concentrated cocaine base case sample on NIR spectra after SNV-1^st^ derivative pre-processing.

 Figure S8. k-Nearest Neighbors distances plots of levamisole (A), lidocaine (B), acetaminophen (C) and ketamine (D) on NIR spectra after SNV-1^st^ derivative pre-processing.

 Figure S9. k-Nearest Neighbors distances plots of amphetamine (E), mephedrone (F), MDMA powder (G) and methamphetamine (H) on NIR spectra after SNV-1^st^ derivative pre-processing.

Table S1. Absolute performance characteristics of the full model and parts of the model after various forms of pre-processing.

Figure S10. kNN distances (A) and correlations (B) plots for a non-matching unknown ‘2C-B’ sample projected on all database spectra.

Table S2. Results of unknown case samples projected on the model and compared with laboratory results. Predicted concentrations between brackets originate from the ANN-BT sub-model, all other concentrations are predictions from the kNN sub-model. Results in red are incorrect. APB = aminopropyl-benzofuran; AMT = alpha-methyltryptamine; DMMC: dimethyl-methcathinone; MDPV: methylenedioxypyrovalerone, 4-FA: 4-fluoroamphetamine.

Table S3. Results of various NPS and drug containing case samples projected on the model and compared with laboratory results.
